# Structures and Applications of Nucleic Acid-Based Micelles for Cancer Therapy

**DOI:** 10.3390/ijms24021592

**Published:** 2023-01-13

**Authors:** Haejoo Kim, Minseok Kwak

**Affiliations:** Department of Chemistry and Industry 4.0 Convergence Bionics Engineering, Pukyong National University, 45 Yongso-ro, Nam-gu, Busan 48513, Republic of Korea

**Keywords:** DNAs, RNAs, amphiphiles, nanoparticles, targeting, chemotherapy, immunotherapy, gene silencing

## Abstract

Nucleic acids have become important building blocks in nanotechnology over the last 30 years. DNA and RNA can sequentially build specific nanostructures, resulting in versatile drug delivery systems. Self-assembling amphiphilic nucleic acids, composed of hydrophilic and hydrophobic segments to form micelle structures, have the potential for cancer therapeutics due to their ability to encapsulate hydrophobic agents into their core and position functional groups on the surface. Moreover, DNA or RNA within bio-compatible micelles can function as drugs by themselves. This review introduces and discusses nucleic acid-based spherical micelles from diverse amphiphilic nucleic acids and their applications in cancer therapy.

## 1. Introduction

**Figure 1:**
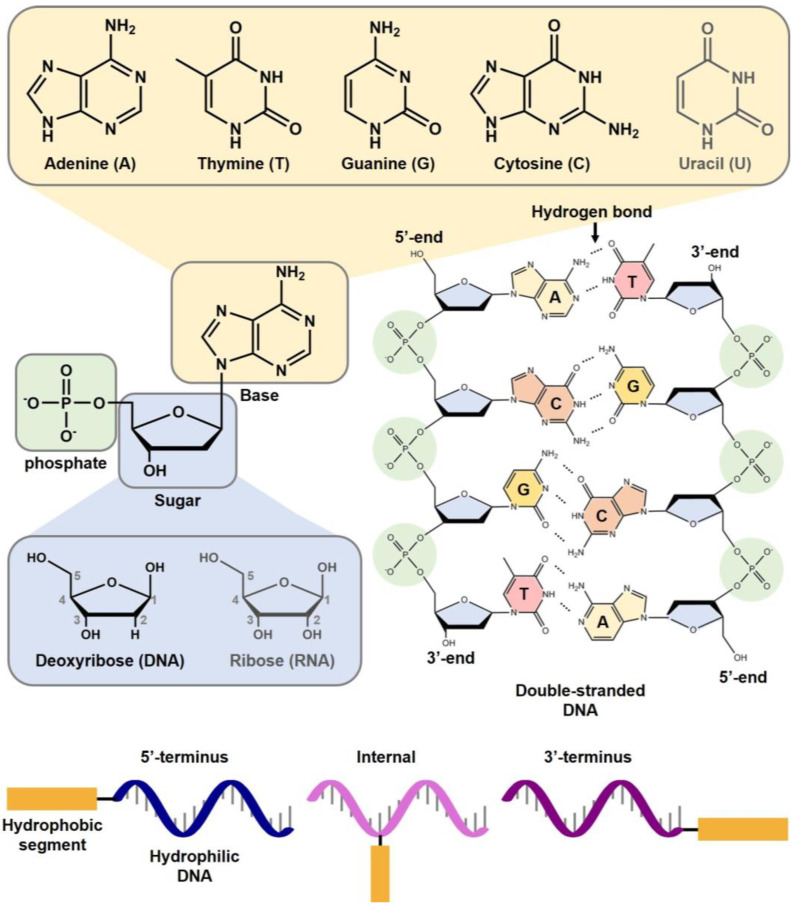
Schematic representation of a nucleotide, double-stranded DNA, and amphiphilic DNA. The nucleotide structure, including a phosphate, a sugar, and a base, is presented on the left. The structural difference between deoxyribose and ribose is shown below the nucleotide structure. The chemical structure of five nucleobases is presented at the top. A detailed structure of double-stranded DNA, composed of two chemically synthesized oligodeoxyribonucleotides, is presented on the right side. The 5’-end and 3’-end indicate the anti-parallel directionality of dsDNA. The dotted lines denote the hydrogen bonds between nucleobases. The positions of hydrophobic segments in amphiphilic DNA are shown at the bottom.

Nucleic acids are biopolymers, which consist of nucleotide monomers. Each nucleotide includes three units: a heterocyclic base, a phosphate group, and a ribose molecule. One after another, nucleosides are linked via a phosphodiester bond forming nucleotides. While ribonucleic acid (RNA) has a 2’-hydroxy group, deoxyribonucleic acid (DNA) has a hydrogen instead at the position. The consecutive nucleotides are named single-stranded (ss) nucleic acid and have directionality such as the 5’ and 3’-ends. DNA’s nucleobases are composed of adenine (A), thymine (T), cytosine (C), and guanine (G), while RNA’s nucleobases have adenine, cytosine, guanine, and uracil (U) instead of thymine. Based on Watson–Crick base-pairing, complementary ssDNA can form an anti-parallel double-stranded DNA (dsDNA). These base-pairs between nucleobases are formed by two hydrogen bonds between adenine and thymine, and three hydrogen bonds between cytosine and guanine (Figure 1) [1,2,3,4].

Nucleic acids are attractive building blocks for nanosized two- and three-dimensional architectures due to their unique characteristics. Firstly, nucleic acid nanostructures have programmability with a defined sequence and accurate designs because base-pairing between nucleobases is highly specific and predictable despite the simple structural composition of nucleic acids. Secondly, nucleic acids, customized with the desired sequence, can be chemically or biologically synthesized owing to well-established processes and protocols. Also, it is possible to modify nucleotides chemically to create functionalized nucleic acids. These nucleic acid syntheses can provide a variety of well-designed nucleic acid structures. Thirdly, such nanomaterials are capable of control at a subnanometer level of precision due to their building units and chemical functionalization. Finally, the conformational change of the nanostructure can be induced depending on temperature, pH, ion, and enzymatic conditions. Therefore, nucleic acid nanotechnology for building nanobiomaterials has been successfully developed and constructed [5,6,7,8,9].

Nucleic acid nanostructures are widely utilized in different fields, especially biomedical applications, due to their excellent biodegradability, biocompatibility, and versatile functional abilities. The sequence specificity and remarkable programmability of nucleic acid nanostructures allow the material to load and deliver biological molecules such as nucleic acids, peptides, and proteins. Based on these advantages, nucleic acid nanostructures have been applied to recognize target cells, control enzymatic activity, and gene silencing for cancer diagnostics and therapeutics [10,11,12,13,14].

Cancer is considered one of the primary causes of mortality throughout the world. The reason for complex cancer therapy is cancer’s ability to evade the immune system, proliferate rapidly and uncontrollably, and metastasize to different tissues. Thus, early diagnosis and appropriate treatments are necessary to improve the survival rate of cancer patients. It is also essential to develop cancer therapeutic technology to reduce side effects and the risk of recurrence [15,16]. Although various therapeutic agents used clinically for cancer treatment have been developed, these small molecule drugs are limited in use due to their high hydrophobicity and low specificity, sensitivity, and bioavailability. In several in vitro and in vivo conditions, many cancer treatment agents have been shown to be highly potent. However, when applied to cancer patients in clinical trials, the agents, due to their low targeting ability and cell membrane permeability, require a higher therapeutic concentration, which results in severe or mortal side effects. Furthermore, hydrophobic and poor soluble agents require a solubilizing mixture containing cytotoxic adjuvants. In addition, most small molecule agents are rapidly eliminated in the liver and kidneys. Therefore, drug delivery systems transporting hydrophobic drugs are beneficial to overcome these disadvantages and improve anti-cancer efficiency [17,18,19].

Polymeric micelles, composed of a hydrophobic part on the core and a hydrophilic part on the shell in aqueous media, are made by the self-assembly of amphiphilic polymers. The morphology and sizes of micelle structures vary according to the hydrophobic volume, concentration, pH value, temperature, and solvent. Its micelle core can load low solubility and highly hydrophobic molecules. Also, the micelle formulations impart stability to therapeutic agents and accumulation in the target site. The internalization of agents in micelle to cancer cells is enhanced compared to free agents by the micelle system’s appropriate hydrophilic and hydrophobic balance. In addition, the surface of micelles can be chemically conjugated with various cancer ligands to offer specific therapeutic molecular targeting. As a result, the micelle-based delivery system is a suitable strategy in cancer therapy [20,21,22].

**Figure 2:**
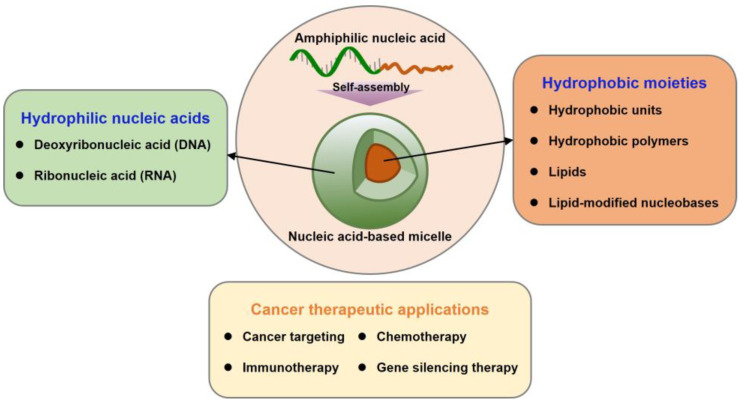
Outline of this review dealing with nucleic acid micelles.

In light of such a strategy, nucleic acid amphiphiles can form biocompatible micelles at the nanoscale. Fundamentally, nucleic acid-based micelles have the unique ability of polymeric micelles, such as encapsulating hydrophobic molecules into their core and functionalizing on their surface. In contrast to polymeric micelles, nucleic acid micelles can introduce therapeutic agents by straightforward base-pairing. In other words, nucleic acid-based micelles can be decorated with various functional moieties via bottom-up construction. In addition, the significant components of the cell membrane are phospholipids and cholesterol. Accordingly, amphiphilic nucleic acids as micelle components improve cell permeability due to their similar structure to the cell membrane [23,24]. Nucleic acid-based micelles, with their biocompatibility, structural programmability, stable loading possibilities, and versatile functionality, are attractive carriers for the delivery of cancer therapeutic agents. This review comprehensively introduces nucleic acid-based micelles with diverse compositions and their applications in cancer therapy (Figure 2).

## 2. Nucleic Acid-Based Micelles

Nucleic acid-based micelles basically require amphiphilic structures consisting of hydrophilic and hydrophobic segments. Since pristine nucleic acids are negatively charged hydrophilic biopolymers, the hydrophobic segment is provided by chemical synthesis within the nucleic acid sequence at either the 5’- or 3’-terminus. In other words, distinct hydrophobic moieties can be introduced at a wide range of positions on nucleic acids, allowing for the production of prescribed amphiphile nucleic acids within the dimensions of 100 nm or smaller (Figure 1).

### 2.1. DNA Micelles

For the synthesis of amphiphilic DNA, two synthetic strategies are used. Firstly, organic-phase coupling is a solution-based synthesis method that utilizes a surfactant-DNA complex for DNA modification in the organic phase [25]. The other process is solid-phase coupling. Automated oligo synthesizers perform sequential couplings of the hydrophobic moieties in addition to coupling cycles of standard nucleobases on solid supports, either controlled pore glass or poly(styrene), using customized hydrophobic phosphoramidites [26,27]. These syntheses allow for versatile functionalization, various modifications, and high reproducibility.

**Figure 3:**
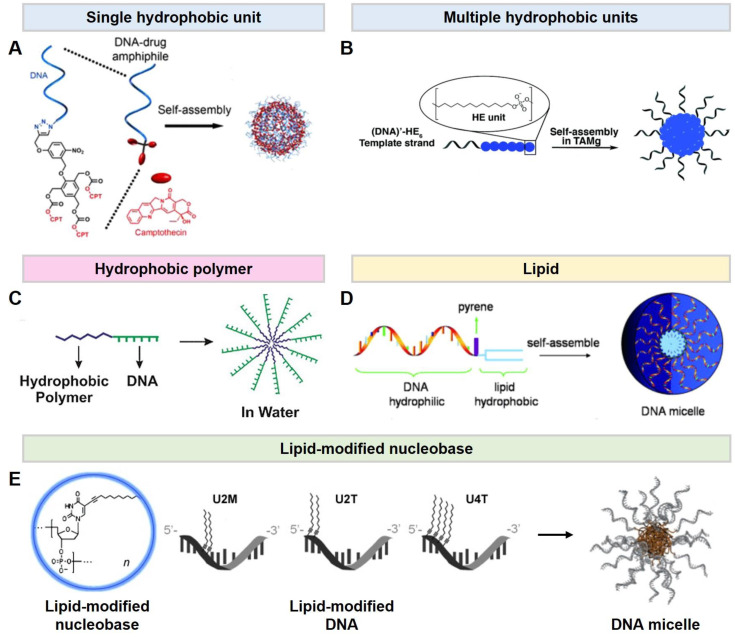
Different types of DNA micelles. (**A**,**B**). Hydrophobic molecule-DNA micelles. (**A**). Schematics of the DNA–Drug (Camptothecin; CPT) micelles assembled from DNA–Drug amphiphiles. Reproduced from ref. [28]. (**B**). Self-assembly of DNA amphiphiles into micelles in aqueous media. Reproduced from ref. [29]. (**C**). A hydrophobic polymer-DNA micelle. Self-assembly of DNA diblock copolymer amphiphiles into a micelle. Reproduced from ref. [34]. (**D**). Lipid-DNA micelle. The design of a lipid-DNA amphiphile and the assembly of DNA micelles. Reproduced from ref. [36]. (**E**). A lipid-modified DNA micelle. Schematic representation of lipid-modified DNA amphiphiles. The number and position of the lipid-modified nucleobase are indicated as the number and M/T, respectively. Reproduced from refs. [44,45].

In this section, micelle-forming DNA materials are categorized by four hydrophobic constituents. To prepare DNA amphiphiles, there are four representative types of hydrophobic moiety, linked at either the 5’- or 3’-end of a DNA strand. (1) One type of amphiphilic DNA is composed of a single or multiple hydrophobic units on oligomeric DNA. The hydrophobic unit is an anti-cancer drug covalently connected to a DNA via a click reaction. Then, the amphiphilic DNA (DNA-drug) can self-assemble into a micelle and carry the drugs simultaneously (Figure 3A) [28]. (2) The other type is a hydrophobic monomer using aliphatic diol-phosphoramidites. The number and position of the hydrophobic units in the hydrophobic sequence are controlled in the solid-phase synthesis steps. Adaptably, this type of DNA amphiphiles can readily tune the lengths of hydrophobic moiety and physical properties, accordingly (Figure 3B) [29]. (3) The third type of commonly used hydrophobic moiety in amphiphilic DNA is hydrophobic polymers including poly(lactic-co-glycolic acid), poly(propylene oxide), and poly(styrene) (Figure 3C) [30,31,32,33,34]. A DNA amphiphile composed of a hydrophobic polymer conjugated to an end of hydrophilic DNA is called a DNA diblock copolymer. Above the critical micelle concentration (CMC), amphiphilic copolymers aggregate forming self-assembled micelle systems due to microphase-separation. The resulting micelles have the unique capability to load hydrophobic drugs or fluorescence dyes in their core. (4) Lastly, lipids are categorized as a hydrophobic motif in DNA amphiphiles. Cholesterol, dipalmitoylphosphatidylethanolamine (DPPE), or a diacyl lipid are connected at the termini of hydrophilic DNA (Figure 3D) [35,36,37,38,39,40,41]. These lipids are covalently conjugated to DNA via a phosphodiester bond. Lipid-conjugated DNA is aggregated to form micelles by the interaction of lipophilic residues. Amphiphilic DNA interacts with the lipid bilayer and has excellent cell permeability due to its similar chemical structure. Furthermore, a hydrophobic moiety can be conjugated at the nucleobase instead of at the DNA termini (Figure 3E) [42,43,44]. Lipid-modified ssDNA contains a hydrophobically modified nucleobases synthesized with a dodec-1-yne group at the C5 position of uracil. The lipid-modified DNA is prepared through solid-phase DNA synthesis with 5-(dodec-1-ynyl)uracil phosphoramidite. The lipophilic nucleobases can be located with an arbitrary number and positions. In aqueous media, the lipid-modified ssDNA can self-assemble into spherical micelles, composed of a hydrophilic DNA corona and a lipophilic DNA core, via microphase-separation. The hydrophobicity of lipid-modified DNA and the thermodynamical stability of its micelle depend on the sequences such as the location and number of hydrophobic nucleobases. The hybridization between a DNA based micelle and its complementary DNA allows for sequence-specific functionalization. Fortunately, the lipophilic group does not sterically interfere with DNA hybridization. The functional molecules, such as fluorescence dye, anti-cancer agents, and cancer ligands, connected at the terminal of the complementary DNA are exposed onto the surface of micelles via base-pairing.

### 2.2. RNA Micelles

**Figure 4:**
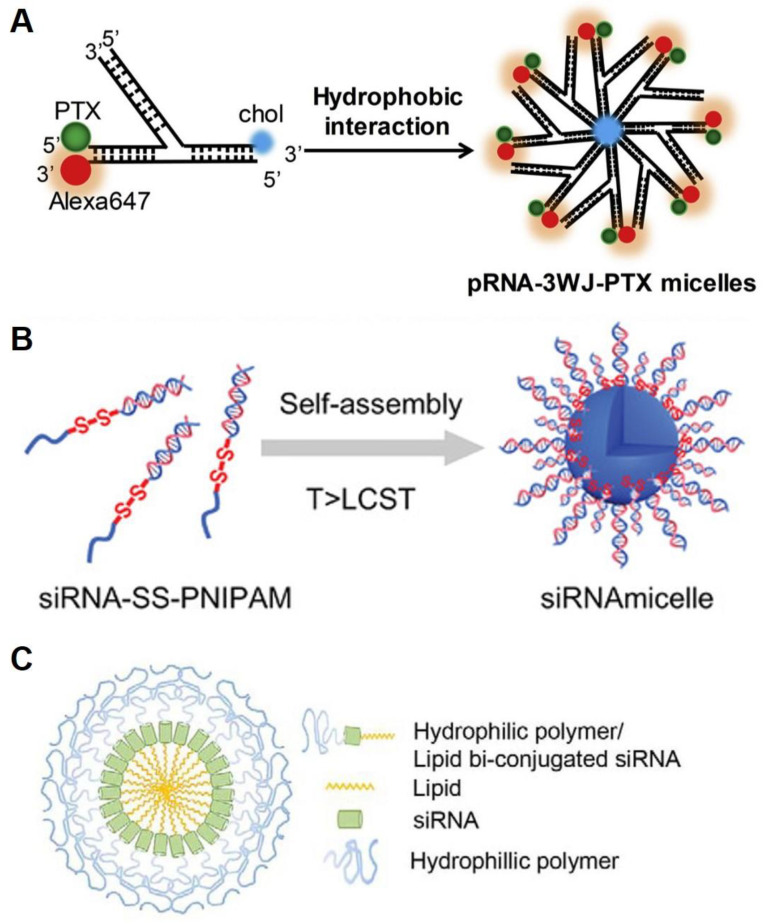
Different types of RNA micelles. (**A**). Schematics of the three-way junction micelles by hydrophobic interaction of cholesterol in aqueous media. Reproduced from ref. [46]. (**B**). Schematic illustration of an RNA micelle. Reproduced from ref. [49]. (**C**). The design of self-assembled micelle inhibitory RNA (SAMiRNA). Reproduced from ref. [50].

In addition to DNA, RNA is also a biopolymer forming versatile micelles. The first amphiphilic RNA reported was based on a three-way junction motif (Figure 4A) [46]. The motif was conjugated with lipophilic moieties, anti-cancer drugs, and fluorescent dyes onto each of the branched ends through RNA solid-phase coupling. The self-assembled RNA micelles, composed of a functionalized RNA corona and a lipophilic core, were regular in shape and size with high thermodynamic stability. The authors showed that the multi-functional RNA micelles exhibited low cytotoxicity, improved cell permeability, and cancer targeting abilities. Commonly, cationic materials in RNA delivery systems are utilized due to their rigid, highly charged nature. However, the cationic macromolecules exhibit potential immunogenicity and toxicity [47,48]. Interestingly, an RNA amphiphile synthesized via click chemistry used a cation-free system (Figure 4B) [49]. The amphiphilic RNA self-assembled, forming a hydrophobic core and a hydrophilic RNA corona of the micelle. The RNA micelles displayed no toxicity associated with charges and the ability to load drugs. Another example consists of a hydrophobic synthetic lipid and a hydrophilic polymer (polyethylene glycol; PEG) at each end of an RNA strand (Figure 4C) [50]. In an aqueous solution, the modified RNA spontaneously forms a micellar structure with three layers, which consists of RNA surrounded by a hydrophobic lipid core and a hydrophilic polymer shell.

## 3. Cancer Therapeutic Applications

Currently, various therapeutic agents are used in targeting cancer in chemotherapy, immunotherapy, and gene therapy for cancer treatment. Nucleic acid-based micelles allow for the loading of anti-cancer drugs into the hydrophobic core and the incorporation of therapeutic agents on the micelle’s surface to target cancer cells, which enhances the retention time and reduces side effects. Here, we chose representative studies applied to cancer therapy using nucleic acid-base micelles (Table 1).

### 3.1. Cancer Targeting

**Figure 5:**
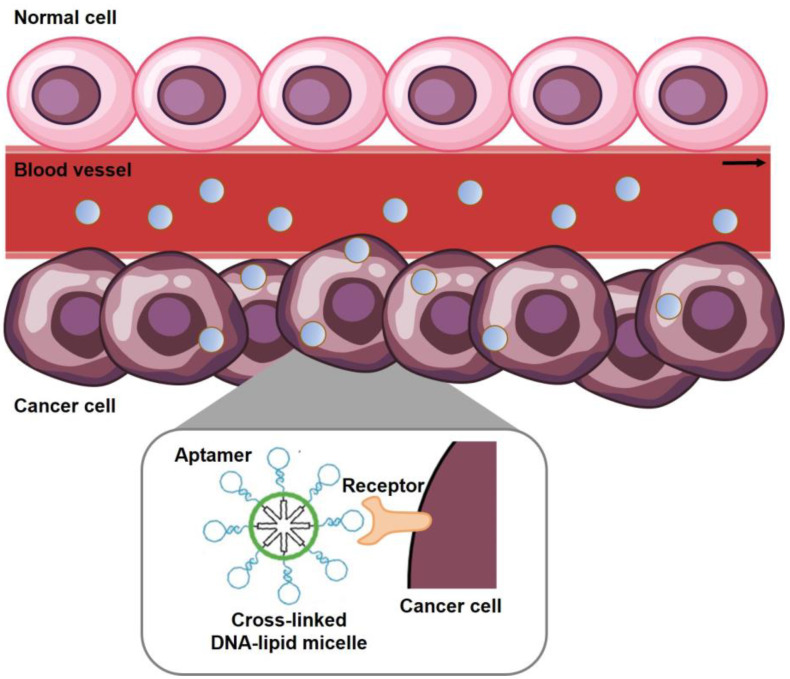
Schematic illustration shows the aptamer-nucleic acid micelle targeting cancer cells through aptamer-receptor recognition (reproduced from ref. [52]).

Anti-cancer drugs affect not only malignant tumor cells but also normal cells, which leads to serious side effects. Thus, it is necessary to develop an accurate targeting system for recognizing cancer cells only. Targeting agents are classified as small molecules (vitamins), aptamers, peptides (fragments of proteins), and proteins (antibodies) [57,58,59]. Among the agents, aptamers can selectively bind to specific receptors of cancer cells and have the potential to induce effective drug delivery to the target cells. The advantages of aptamers are their small size, low immunogenicity, and easy penetration [60,61,62]. Also, the hydrophobic moiety can be covalently attached to the end of aptamers, that are short oligonucleotides with hydrophilic moiety, to synthesize amphiphile nucleic acid. In addition, the amphiphilic aptamers can build a nucleic acid-based micelle structure [51,52]. In Figure 5, the sgc8 aptamer, which specifically recognizes the PTK7 receptor on CCRF-CEM cells (T-cell acute lymphoblastic leukemia cell line), is used in the aptamer-DNA block copolymer. The UV-induced cross-linking of methacrylamide, the linker between the hydrophobic moiety and the aptamer, enhances the structural stability of aptamer-micelles. This aptamer-micelle system promotes the recognition of cancer cells and improves selective targeting ability in CCRF-CEM cells [52].

### 3.2. Chemotherapy

Chemotherapy is a drug treatment that utilizes cytotoxic chemicals to remove cancer cells and inhibit cancer growth. Although many anti-cancer drugs are generally used in cancer treatment, there are several concerns. Most drugs have poor water solubility, low cell permeability, and indiscriminate cytotoxicity to cancer cells as well as normal cells [63,64,65]. Therefore, nucleic acid-based micelles can be used to overcome the drawbacks of chemotherapy by encapsulating drugs to enhance their solubility, permeability, and targeting abilities [66,67,68].

**Figure 6:**
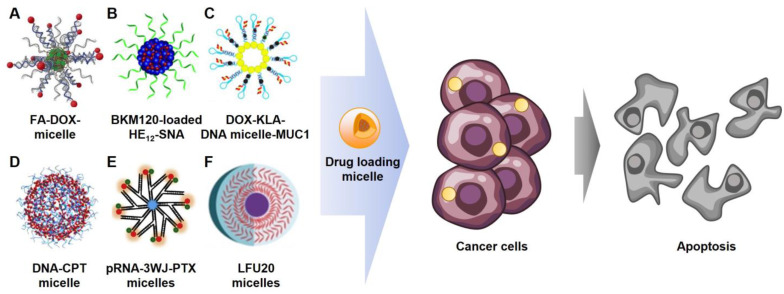
Diverse chemotherapeutic micelles. (**A**). Folic acid (FA) functionalized and doxorubicin (DOX) loaded micelle (ref. [45]) (**B**). Buparlisib (BKM120) loaded micelle (ref. [53]) (**C**). Mucine1 aptamer (anti-MUC1) functionalized, and (KLAKLAK)2 (KLA) and DOX loaded micelle (ref. [39]) (**D**). Camptothecin (CPT) loaded micelle (ref. [28]) (**E**). Paclitaxel (PTX) and anti-MUC1 functionalized micelle (ref. [46]) (**F**). Floxuridine micelle (ref. [54]).

Generally, micelle systems can load hydrophobic drugs into the micelle core via hydrophobic interactions (Figure 6A,B) [45,53]. In Figure 6C, doxorubicin (DOX) was captured within the nucleic acid structure of the micelle by intercalation [39]. The intercalation method enables the loading of various anti-cancer drugs, such as DOX, daunorubicin, and mitoxantrone [69,70], by π-π stacking with contributions from van der Waals forces and hydrophobic interactions [71,72,73]. The amphiphilic nucleic acids in Figure 6D,E are covalently conjugated with hydrophobic drugs, CPT and PTX, respectively, at the end of the hydrophilic nucleic acid to improve drug solubility [28,46]. Then, the drugs can be exposed on the micelle surface or located in the micelle core by self-assembling amphiphilic nucleic acids. Additionally, amphiphilic nucleic acids can be chemotherapeutic agents themselves. A cancer-therapeutic nucleoside analog, fluorouridine, was employed in synthesizing DNA connected with a lipophilic moiety [74,75,76]. As a result, the amphiphilic nucleic acids can spontaneously form a micelle containing anti-cancer drugs on its shell (Figure 6F) [54].

Furthermore, targeting agents of cancer cells could be incorporated on the surface of micelles. For example, a micelle equipped with folic acid (FA), covalently linked to the complementary DNA by base-pairing (Figure 6B). The folic acid-micelles could then bind to folate receptors, which are highly expressed in MCF-7 cells (human breast adenocarcinoma cell lines). In Figure 6E, a mucine1 aptamer (anti-MUC1) was conjugated to the micelle for targeting the MUC1 receptors overexpressed on the surface of cancer cells. These targeted drug delivery systems exhibited enhanced cell permeability and cell-uptake efficiency.

### 3.3. Immunotherapy

Immunotherapy is a cancer treatment based on using the natural immune system to eliminate cancer cells by targeting cancer-specific antigens. Immunotherapeutic agents are immune adjuvants used to induce an antigen-specific immune response [77,78,79]. One of the immune adjuvants is CpG oligodeoxyribonucleotides (CpG ODNs), including unmethylated cytosine-phosphate-guanine dinucleotide sequences. The CpG ODN is recognized by toll-like receptor 9 (TLR9). Then, the recognition induces the activation of dendritic cells, enhances cancer antigen presentation, and promotes an anti-cancer immune response [80,81,82]. However, the usage of CpG ODNs has challenges, such as easy degradation by nucleases, poor cell internalization, and low biodistribution. In consideration of these challenges, nucleic acid-based micelles are utilized to deliver CpG ODNs [83,84,85].

Herein, nucleic acid-based micelles for immunotherapy decorated with CpG ODN are introduced. Since CpG ODN consists entirely of nucleic acids, it can be used as an immune adjuvant. Banga et al. proposed an immunotherapeutic micelle containing CpG ODN. In a nucleic acid amphiphile, CpG ODN is directly attached to a phospholipid. The micelle, incorporated with an immune adjuvant on the surface, is made by self-assembly of amphiphilic CpG ODNs. This immunotherapeutic micelle system showed enhanced stability in a physiological environment, rapid cellular internalization, and effective immune activation compared to pristine ODN [35].

**Figure 7:**
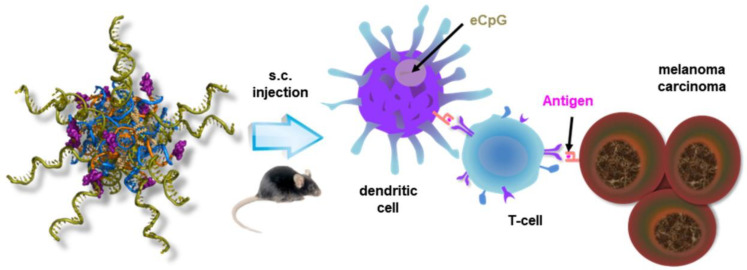
Schematic representation of nucleic acid-based micelle’s immunotherapy. Reproduced from ref. [55].

In another example in 2019, Jin et al. constructed a nucleic acid-based micelle decorated with CpG ODN by the hybridization of nucleic acids. Amphiphilic nucleic acids, composed of a lipid-modified nucleobase, form scaffold micelles as carriers. Immune adjuvant CpG ODN is connected to a complementary sequence of scaffold micelles. Then, multiple CpG ODNs are easily incorporated onto the surface of micelles via base-pairing. In the same way, a peptide epitope (smaller sized antigen fragment) is prepared and incorporated. This micelle system demonstrated effective co-delivery of immune adjuvants and antigens to the spleen and lymph nodes, the induction of an antigen-specific immune response, and the inhibition of cancer growth together with anti-metastasis effects (Figure 7) [55].

### 3.4. Gene Silencing Therapy

Gene silencing therapy is a cancer treatment approach based on RNA interference (RNAi) that regulates the expression of genes associated with cancer cells. In gene silencing therapy, different types of RNAi molecules such as microRNAs (miRNAs), siRNAs, and short hairpin RNAs (shRNAs) are used [86,87,88]. However, gene silencing treatment is challenged by the following problems. RNAs are not stable in a biological environment due to nuclease-induced degradation and hydrolysis. Also, the RNAi molecule’s physiochemical properties of high molecular weight and negative charges make it difficult to reach the target cells [89,90,91]. Thus, the RNA delivery micelle system can be adapted to overcome these problems [92,93,94].

Yin et al. proposed RNA-based micelles to deliver anti-miR21 to cancer cells. MicroRNA21 (miR21) is one of the cancer-promoting miRNAs, short ssRNA molecules associated with gene expression, targeting cancer suppressor genes related to cell proliferation, invasion, and apoptosis [95,96]. The RNA-based micelles, stable in a wide range of temperatures, pH, and RNase environments, are made by the phi29 packaging RNA three-way junction (pRNA-3WJ) scaffold. Each rim of the junction is conjugated with hydrophobic cholesterol of a micelle core, anti-miR21, and folate, respectively. Folate decorating micelles can enhance targeting and internalization into KB cells (epithelial carcinoma cells). The multifunctional micelle can carry anti-miR21 to its target, induce apoptosis, and prevent the proliferation of the cancer [56].

Another siRNA delivery micelle was constructed by Jiang et al. for the treatment of glioblastoma (GBM, malignant brain tumor). The siRNA, a short double-stranded RNA with a length of 19–23 base pairs, is incorporated into the RNA-induced silencing complex (RISC) to cleave and degrade a target messenger RNA (mRNA) resulting in interference with specific gene expression [97,98,99]. This siRNA is a gene silencing agent in cancer therapy that can regulate tumor genes and be conjugated with poly(*N*-isopropylacrylamide) of the hydrophobic moiety to form micelles. Due to the dense negative charges and surrounding counterion shells, the siRNA-micelle exhibits resistance against nuclease degradation and delivery to cancer cells through scavenger receptors (SRs, highly expressed on cancer cells) mediated endocytosis. The siRNA-micelles show significant accumulation in brain tumor cells and tumor growth inhibition. Furthermore, the micelle system induces notable synergistic effects in cancer therapy via loading temozolomide (TMZ, brain anti-tumor drug) into the micelle core [49].

## 4. Conclusions and Outlook

Nucleic acids have unique structural and chemical properties, which are possible to create various nanostructures of desired sizes and shapes. The nucleic acid-based nanostructures have been developed and utilized in a wide range of applications. Notably, in biomedical applications, these nanostructures are used as the delivery platform due to their major advantages such as biocompatibility, low cytotoxicity, biodegradability, and multi-functional capacity. Despite the developments in cancer treatment, the usage of therapeutic agents is limited due to non-specific cytotoxicity, uneven dispersion, and multi-drug resistance. Accordingly, a nucleic acid-based micelle is one of the most promising nanostructures to overcome these limitations. The hydrophobic core of the micelle can load anti-cancer drugs to enhance poor solvent compatibilities, to prevent release at unwanted places, and to suppress resistance to the drugs. Simultaneously, the hydrophilic shell can be functionalized with ligands to offer cancer cell targeting and facile internalization. In addition, the similar structure of amphiphilic nucleic acids, components of the micelle, with the cell membrane improves cell permeability and prevents severe side effects resulting from a higher dose due to low permeability.

Nucleic acid-based micelles, composed of a hydrophobic core and a hydrophilic corona, are constructed from amphiphilic nucleic acids via self-assembly. Since amphiphilic nucleic acids are based on hydrophilic nucleic acids, the hydrophobicity should be imparted through chemical synthesis. Different types of hydrophobic molecules are used and classified as single hydrophobic molecules, polymers, lipids, and lipid-modified nucleobases. The lengths, shapes, and sequences of hydrophilic nucleic acids can be controlled by automated synthesis processes. Depending on the variety of selecting hydrophobic and hydrophilic moieties, different morphologies of micelle structures can be constructed. Functionalized nucleic acid-based micelles are applied as different types of cancer treatment. For example, oligonucleotide aptamers are used as hydrophilic nucleic acids to impart the ability of cancer targeting. Also, a variety of chemotherapeutic drugs are encapsulated into the hydrophobic core of the micelle. In immunotherapeutic applications, immune adjuvant CpG ODNs consisting entirely of nucleic acids become a hydrophilic moiety or are incorporated on the surface of the micelle via base-pairing. Gene silencing miRNA and siRNA are also used as therapeutic agents in micelles. The decisive advantage of the micelle is that it is possible to multi-functionalize in one micelle. Multi-functional micelles, such as the combination of chemotherapy-targeting cancer and gene silencing therapy-targeting cancer, provide more enhanced anti-cancer efficacy than mono-functional micelles by additive and synergistic manners [100,101,102]. As a result of these advantages, nucleic acid-based micelle systems are considered a promising anti-cancer delivery strategy and have become increasingly important in cancer therapy.

Although nucleic acid-based materials are state-of-the-art technology broadening the applications in vaccines and beyond, such as the SARS-CoV-2 virus [103,104,105,106], the current formulation, namely lipid nanoparticle, has a low mRNA loading capacity (<5%) compared to the extent of its carrier components [107,108,109,110]. Therefore, there is room to develop innovative delivery methods using nucleic acid-based materials including micellar systems. We envision that such techniques using RNA and DNA biopolymers should have minimal amounts of immunogenic moieties, the ability to control the release of mRNAs, and well-defined stable structures to overcome current issues like safety, storage, and multiple vaccinations.

Over 20 years or more, we have witnessed many studies of synthetic methods for nucleic acid micelles and their proofs-of-concept in cancer therapy, which is thanks to the splendid advances in recent gene editing and mRNA technologies. Self-assembled DNA and RNA nanostructures, including micelles, face new challenges to flourish. In therapeutics, it is time to pursue clinical trials, and the entire biomedical DNA nanotechnology field will broaden its utilization even further.

## Figures and Tables

**Table 1:** Examples of various nucleic acid-based micelles for cancer therapy.

Nucleic Acid	Hydrophobic Moiety	Size (nm)	Therapeutic Agent	Therapy	Cell or Tumor Type	Ref.
DNA	Diacyl chain	68 ± 13	TDO5 aptamer	Cancer targeting	Ramos cells	[51]
DNA	Diacyl lipid	17 ± 2	sgc8 aptamer	Cancer targeting	CCRF-CEM cells	[52]
DNA	PPO	10.8 ± 2.2	Doxorubicin (Dox), Folic acid (FA)	Chemotherapy Cancer targeting	Caco-2 cells	[45]
DNA	Dodecane	11.8 ± 0.4	BKM120	Chemotherapy	HeLa cells	[53]
DNA	Cholesterol	371.3 ± 3.1	DOX, anti-MUC1, KLA peptide	Chemotherapy Cancer targeting	MCF-7 cells, C26-tumor	[39]
DNA	CPT	32 ± 13	CPT	Chemotherapy	SK-BR-3 cells	[28]
RNA	Cholesterol	118.7 ± 14.50	Paclitaxel (PTX)	Chemotherapy	Human KB cells, KB tumor	[46]
DNA	Octadecyl chain	43	Fluorouracil	Chemotherapy	HeLa cells	[54]
DNA	DPPE lipid	25 ± 5	ODN 1826, ODN 7909	Immunotherapy	HEK-Blue™-mTLR9 cells, Ramos-Blue cells	[35]
DNA	Dodec-1-ynyl uracil	10.10 ± 0.68	ODN 1826, OVA antigen	Immunotherapy	B16-OVA cells, B16 melanoma, CT-26 carcinoma	[55]
RNA	Cholesterol	21.51 ± 6.136	anti-miR21, folate	Gene silencing therapy Cancer targeting	KB cells, HT29 cells, KB tumor	[56]
RNA	poly(*N*-isopropylacrylamide)	40~150	siPLK1, temozolomide (TMZ)	Gene silencing therapy Chemotherapy	U87MG cells, U251 cells	[49]

## Data Availability

Not applicable.

## References

[1] Watson J.D., Crick F.H.C. (1953). Molecular Structure of Nucleic Acids: A Structure for Deoxyribose Nucleic Acid. Nature.

[2] Neidle S., Sanderson M. (2021). Principles of Nucleic Acid Structure.

[3] Pedersen R., Marchi A.N., Majikes J., Nash J.A., Estrich N.A., Courson D.S., Hall C.K., Craig S.L., LaBean T.H., Bhushan B., Luo D., Schricker S.R., Sigmund W., Zauscher S. (2014). Properties of DNA. Handbook of Nanomaterials Properties.

[4] Watson J.D., Crick F.H. (1953). A Structure for Deoxyribose Nucleic Acid.

[5] Jaeger L., Chworos A. (2006). The architectonics of programmable RNA and DNA nanostructures. Curr. Opin. Struct. Biol..

[6] Ramezani H., Dietz H. (2020). Building machines with DNA molecules. Nat. Rev. Genet..

[7] Seeman N.C., Sleiman H.F. (2017). DNA nanotechnology. Nat. Rev. Mater..

[8] Gothelf K.V., LaBean T.H. (2005). DNA-programmed assembly of nanostructures. Org. Biomol. Chem..

[9] Brucale M., Zuccheri G., Samorì B. (2006). Mastering the complexity of DNA nanostructures. Trends Biotechnol..

[10] Sun Q., Han Y., Yang Y., de la Fuente J.M., Cui D., Wang X. (2020). Application of DNA nanostructures in cancer therapy. Appl. Mater. Today.

[11] Wang D.-X., Wang J., Wang Y.-X., Du Y.-C., Huang Y., Tang A.-N., Cui Y.-X., Kong D.-M. (2021). DNA nanostructure-based nucleic acid probes: Construction and biological applications. Chem. Sci..

[12] Nicolson F., Ali A., Kircher M.F., Pal S. (2020). DNA Nanostructures and DNA-Functionalized Nanoparticles for Cancer Theranostics. Adv. Sci..

[13] Bujold K.E., Lacroix A., Sleiman H.F. (2018). DNA Nanostructures at the Interface with Biology. Chem.

[14] Lacroix A., Sleiman H.F. (2021). DNA Nanostructures: Current Challenges and Opportunities for Cellular Delivery. ACS Nano.

[15] (2021). Advancing Cancer Therapy. Nat. Cancer.

[16] (2020). The global challenge of cancer. Nat. Cancer.

[17] Qin S.-Y., Zhang A.-Q., Cheng S.-X., Rong L., Zhang X.-Z. (2017). Drug self-delivery systems for cancer therapy. Biomaterials.

[18] Sun T., Zhang Y.S., Pang B., Hyun D.C., Yang M., Xia Y. (2021). Engineered nanoparticles for drug delivery in cancer therapy. Nanomaterials and Neoplasms.

[19] Peer D., Karp J.M., Hong S., Farokhzad O.C., Margalit R., Langer R. (2020). Nanocarriers as an emerging platform for cancer therapy. Nano-Enabled Medical Applications.

[20] Shin D.H., Tam Y.T., Kwon G.S. (2016). Polymeric micelle nanocarriers in cancer research. Front. Chem. Sci. Eng..

[21] Gong J., Chen M., Zheng Y., Wang S., Wang Y. (2012). Polymeric micelles drug delivery system in oncology. J. Control. Release.

[22] Aliabadi H.M., Lavasanifar A. (2006). Polymeric micelles for drug delivery. Expert Opin. Drug Deliv..

[23] Qiu L., Zhang T., Jiang J., Wu C., Zhu G., You M., Chen X., Zhang L., Cui C., Yu R. (2014). Cell Membrane-Anchored Biosensors for Real-Time Monitoring of the Cellular Microenvironment. J. Am. Chem. Soc..

[24] Huo S., Li H., Boersma A.J., Herrmann A. (2019). DNA Nanotechnology Enters Cell Membranes. Adv. Sci..

[25] Liu K., Zheng L., Liu Q., de Vries J.W., Gerasimov J.Y., Herrmann A. (2014). Nucleic Acid Chemistry in the Organic Phase: From Functionalized Oligonucleotides to DNA Side Chain Polymers. J. Am. Chem. Soc..

[26] Caruthers M.H. (1985). Gene Synthesis Machines: DNA Chemistry and Its Uses. Science.

[27] Kosuri S., Church G.M. (2014). Large-scale de novo DNA synthesis: Technologies and applications. Nat. Methods.

[28] Tan X., Li B.B., Lu X., Jia F., Santori C., Menon P., Li H., Zhang B., Zhao J.J., Zhang K. (2015). Light-Triggered, Self-Immolative Nucleic Acid-Drug Nanostructures. J. Am. Chem. Soc..

[29] Trinh T., Chidchob P., Bazzi H.S., Sleiman H.F. (2016). DNA micelles as nanoreactors: Efficient DNA functionalization with hydrophobic organic molecules. Chem. Commun..

[30] Zhang C., Hao L., Calabrese C.M., Zhou Y., Choi C.H.J., Xing H., Mirkin C.A. (2015). Biodegradable DNA-Brush Block Copolymer Spherical Nucleic Acids Enable Transfection Agent-Free Intracellular Gene Regulation. Small.

[31] Alemdaroglu F.E., Ding K., Berger R., Herrmann A. (2006). DNA-Templated Synthesis in Three Dimensions: Introducing a Micellar Scaffold for Organic Reactions. Angew. Chem. Int. Ed..

[32] Wilks T.R., Bath J., de Vries J.W., Raymond J.E., Herrmann A., Turberfield A.J., O’Reilly R.K. (2013). “Giant Surfactants” Created by the Fast and Efficient Functionalization of a DNA Tetrahedron with a Temperature-Responsive Polymer. ACS Nano.

[33] Jeong J.H., Park T.G. (2001). Novel Polymer−DNA Hybrid Polymeric Micelles Composed of Hydrophobic Poly(d,l-lactic-co-glycolic Acid) and Hydrophilic Oligonucleotides. Bioconjug. Chem..

[34] Li Z., Zhang Y., Fullhart P., Mirkin C.A. (2004). Reversible and Chemically Programmable Micelle Assembly with DNA Block-Copolymer Amphiphiles. Nano Lett..

[35] Banga R.J., Meckes B., Narayan S.P., Sprangers A.J., Nguyen S.T., Mirkin C.A. (2017). Cross-Linked Micellar Spherical Nucleic Acids from Thermoresponsive Templates. J. Am. Chem. Soc..

[36] Liu H., Zhu Z., Kang H., Wu Y., Sefan K., Tan W. (2010). DNA-Based Micelles: Synthesis, Micellar Properties and Size-Dependent Cell Permeability. Chem. Eur. J..

[37] Zou J., Jin C., Wang R., Kuai H., Zhang L., Zhang X., Li J., Qiu L., Tan W. (2018). Fluorinated DNA Micelles: Synthesis and Properties. Anal. Chem..

[38] Godeau G., Arnion H., Brun C., Staedel C., Barthélémy P. (2010). Fluorocarbon oligonucleotide conjugates for nucleic acids delivery. MedChemComm.

[39] Charbgoo F., Alibolandi M., Taghdisi S.M., Abnous K., Soltani F., Ramezani M. (2018). MUC1 aptamer-targeted DNA micelles for dual tumor therapy using doxorubicin and KLA peptide. Nanomed. Nanotechnol. Biol. Med..

[40] Godeau G., Staedel C., Barthélémy P. (2008). Lipid-Conjugated Oligonucleotides via “Click Chemistry” Efficiently Inhibit Hepatitis C Virus Translation. J. Med. Chem..

[41] Liu H., Kwong B., Irvine D.J. (2011). Membrane Anchored Immunostimulatory Oligonucleotides for In Vivo Cell Modification and Localized Immunotherapy. Angew. Chem. Int. Ed..

[42] Kim H., Zhang W., Hwang J., An E.-K., Choi Y.K., Moon E., Loznik M., Huh Y.H., Herrmann A., Kwak M. (2021). Carrier-free micellar CpG interacting with cell membrane for enhanced immunological treatment of HIV-1. Biomaterials.

[43] Jin J.-O., Park H., Zhang W., de Vries J.W., Gruszka A., Lee M.W., Ahn D.-R., Herrmann A., Kwak M. (2017). Modular delivery of CpG-incorporated lipid-DNA nanoparticles for spleen DC activation. Biomaterials.

[44] Anaya M., Kwak M., Musser A.J., Müllen K., Herrmann A. (2010). Tunable Hydrophobicity in DNA Micelles: Design, Synthesis, and Characterization of a New Family of DNA Amphiphiles. Chem. Eur. J..

[45] Alemdaroglu F.E., Alemdaroglu N.C., Langguth P., Herrmann A. (2008). DNA Block Copolymer Micelles—A Combinatorial Tool for Cancer Nanotechnology. Adv. Mater..

[46] Shu Y., Yin H., Rajabi M., Li H., Vieweger M., Guo S., Shu D., Guo P. (2018). RNA-based micelles: A novel platform for paclitaxel loading and delivery. J. Control. Release.

[47] Zipkin M. (2020). Big pharma buys into exosomes for drug delivery. Nat. Biotechnol..

[48] Zuckerman J.E., Gritli I., Tolcher A., Heidel J.D., Lim D., Morgan R., Chmielowski B., Ribas A., Davis M.E., Yen Y. (2014). Correlating animal and human phase Ia/Ib clinical data with CALAA-01, a targeted, polymer-based nanoparticle containing siRNA. Proc. Natl. Acad. Sci. USA.

[49] Jiang T., Qiao Y., Ruan W., Zhang D., Yang Q., Wang G., Chen Q., Zhu F., Yin J., Zou Y. (2021). Cation-Free siRNA Micelles as Effective Drug Delivery Platform and Potent RNAi Nanomedicines for Glioblastoma Therapy. Adv. Mater..

[50] Yoon P.O., Park J.W., Lee C.-M., Kim S.H., Kim H.-N., Ko Y., Bae S.J., Yun S., Park J.H., Kwon T. (2016). Self-assembled Micelle Interfering RNA for Effective and Safe Targeting of Dysregulated Genes in Pulmonary Fibrosis. J. Biol. Chem..

[51] Wu Y., Sefah K., Liu H., Wang R., Tan W. (2010). DNA aptamer–micelle as an efficient detection/delivery vehicle toward cancer cells. Proc. Natl. Acad. Sci. USA.

[52] Li X., Figg C.A., Wang R., Jiang Y., Lyu Y., Sun H., Liu Y., Wang Y., Teng I.-T., Hou W. (2018). Cross-Linked Aptamer–Lipid Micelles for Excellent Stability and Specificity in Target-Cell Recognition. Angew. Chem. Int. Ed..

[53] Bousmail D., Amrein L., Fakhoury J.J., Fakih H.H., Hsu J.C.C., Panasci L., Sleiman H.F. (2017). Precision spherical nucleic acids for delivery of anticancer drugs. Chem. Sci..

[54] Jin C., Zhang H., Zou J., Liu Y., Zhang L., Li F., Wang R., Xuan W., Ye M., Tan W. (2018). Floxuridine Homomeric Oligonucleotides “Hitchhike” with Albumin In Situ for Cancer Chemotherapy. Angew. Chem. Int. Ed..

[55] Jin J.-O., Kim H., Huh Y.H., Herrmann A., Kwak M. (2019). Soft matter DNA nanoparticles hybridized with CpG motifs and peptide nucleic acids enable immunological treatment of cancer. J. Control. Release.

[56] Yin H., Wang H., Li Z., Shu D., Guo P. (2019). RNA Micelles for the Systemic Delivery of Anti-miRNA for Cancer Targeting and Inhibition without Ligand. ACS Nano.

[57] Rosenblum D., Joshi N., Tao W., Karp J.M., Peer D. (2018). Progress and challenges towards targeted delivery of cancer therapeutics. Nat. Commun..

[58] Dai L., Liu J., Luo Z., Li M., Cai K. (2016). Tumor therapy: Targeted drug delivery systems. J. Mater. Chem. B.

[59] Mun E.J., Babiker H.M., Weinberg U., Kirson E.D., Von Hoff D.D. (2018). Tumor-Treating Fields: A Fourth Modality in Cancer Treatment. Clin. Cancer Res..

[60] Kim M., Kim D.-M., Kim K.-S., Jung W., Kim D.-E. (2018). Applications of Cancer Cell-Specific Aptamers in Targeted Delivery of Anticancer Therapeutic Agents. Molecules.

[61] Gao F., Yin J., Chen Y., Guo C., Hu H., Su J. (2022). Recent advances in aptamer-based targeted drug delivery systems for cancer therapy. Front. Bioeng. Biotechnol..

[62] Zhang Y., Hong H., Cai W. (2011). Tumor-Targeted Drug Delivery with Aptamers. Curr. Med. Chem..

[63] Anand U., Dey A., Chandel A.K.S., Sanyal R., Mishra A., Pandey D.K., De Falco V., Upadhyay A., Kandimalla R., Chaudhary A. (2022). Cancer chemotherapy and beyond: Current status, drug candidates, associated risks and progress in targeted therapeutics. Genes Dis..

[64] Chabner B.A., Roberts T.G. (2005). Chemotherapy and the war on cancer. Nat. Rev. Cancer.

[65] Schirrmacher V. (2019). From chemotherapy to biological therapy: A review of novel concepts to reduce the side effects of systemic cancer treatment (Review). Int. J. Oncol..

[66] Li Z., Tan S., Li S., Shen Q., Wang K. (2017). Cancer drug delivery in the nano era: An overview and perspectives (Review). Oncol. Rep..

[67] Senapati S., Mahanta A.K., Kumar S., Maiti P. (2018). Controlled drug delivery vehicles for cancer treatment and their performance. Signal Transduct. Target. Ther..

[68] Dang Y., Guan J. (2020). Nanoparticle-based drug delivery systems for cancer therapy. Smart Mater. Med..

[69] Nussbaumer S., Bonnabry P., Veuthey J.-L., Fleury-Souverain S. (2011). Analysis of anticancer drugs: A review. Talanta.

[70] Huang X., Blum N.T., Lin J., Shi J., Zhang C., Huang P. (2021). Chemotherapeutic drug–DNA hybrid nanostructures for anti-tumor therapy. Mater. Horiz..

[71] Chaires J.B. (2008). Calorimetry and Thermodynamics in Drug Design. Annu. Rev. Biophys..

[72] Agudelo D., Bourassa P., Bérubé G., Tajmir-Riahi H.-A. (2014). Intercalation of antitumor drug doxorubicin and its analogue by DNA duplex: Structural features and biological implications. Int. J. Biol. Macromol..

[73] Jawad B., Poudel L., Podgornik R., Ching W.-Y. (2020). Thermodynamic Dissection of the Intercalation Binding Process of Doxorubicin to dsDNA with Implications of Ionic and Solvent Effects. J. Phys. Chem. B.

[74] Rider B.J., Enna S.J., Bylund D.B. (2007). 5 Fluorodeoxyuridine. xPharm: The Comprehensive Pharmacology Reference.

[75] Longley D.B., Harkin D.P., Johnston P.G. (2003). 5-Fluorouracil: Mechanisms of action and clinical strategies. Nat. Rev. Cancer.

[76] (1995). Efficacy of adjuvant fluorouracil and folinic acid in colon cancer: International Multicentre Pooled Analysis of Colon Cancer Trials (IMPACT) investigators. Lancet.

[77] Schuster M., Nechansky A., Kircheis R. (2006). Cancer immunotherapy. Biotechnol. J..

[78] Waldman A.D., Fritz J.M., Lenardo M.J. (2020). A guide to cancer immunotherapy: From T cell basic science to clinical practice. Nat. Rev. Immunol..

[79] Dougan M., Dranoff G. (2009). Immune Therapy for Cancer. Annu. Rev. Immunol..

[80] Chuang Y.-C., Tseng J.-C., Huang L.-R., Huang C.-M., Huang C.-Y.F., Chuang T.-H. (2020). Adjuvant Effect of Toll-like Receptor 9 Activation on Cancer Immunotherapy Using Checkpoint Blockade. Front. Immunol..

[81] Krieg A.M. (2008). Toll-like receptor 9 (TLR9) agonists in the treatment of cancer. Oncogene.

[82] Dongye Z., Li J., Wu Y. (2022). Toll-like receptor 9 agonists and combination therapies: Strategies to modulate the tumour immune microenvironment for systemic anti-tumour immunity. Br. J. Cancer.

[83] Riley R.S., June C.H., Langer R., Mitchell M.J. (2019). Delivery technologies for cancer immunotherapy. Nat. Rev. Drug Discov..

[84] Chi Q., Yang Z., Xu K., Wang C., Liang H. (2020). DNA Nanostructure as an Efficient Drug Delivery Platform for Immunotherapy. Front. Pharmacol..

[85] Mukalel A.J., Riley R.S., Zhang R., Mitchell M.J. (2019). Nanoparticles for nucleic acid delivery: Applications in cancer immunotherapy. Cancer Lett..

[86] Tian Z., Liang G., Cui K., Liang Y., Wang Q., Lv S., Cheng X., Zhang L. (2021). Insight Into the Prospects for RNAi Therapy of Cancer. Front. Pharmacol..

[87] Mansoori B., Sandoghchian Shotorbani S., Baradaran B. (2014). RNA Interference and its Role in Cancer Therapy. Adv. Pharm. Bull..

[88] Deng Y., Wang C.C., Choy K.W., Du Q., Chen J., Wang Q., Li L., Chung T.K.H., Tang T. (2014). Therapeutic potentials of gene silencing by RNA interference: Principles, challenges, and new strategies. Gene.

[89] Xin Y., Huang M., Guo W.W., Huang Q., Zhang L.z., Jiang G. (2017). Nano-based delivery of RNAi in cancer therapy. Mol. Cancer..

[90] Li D., Gao C., Kuang M., Xu M., Wang B., Luo Y., Teng L., Xie J. (2021). Nanoparticles as Drug Delivery Systems of RNAi in Cancer Therapy. Molecules.

[91] Kara G., Calin G.A., Ozpolat B. (2022). RNAi-based therapeutics and tumor targeted delivery in cancer. Adv. Drug Delivery Rev..

[92] Letsinger R.L., Zhang G.R., Sun D.K., Ikeuchi T., Sarin P.S. (1989). Cholesteryl-conjugated oligonucleotides: Synthesis, properties, and activity as inhibitors of replication of human immunodeficiency virus in cell culture. Proc. Natl. Acad. Sci. USA.

[93] Ezzat K., Aoki Y., Koo T., McClorey G., Benner L., Coenen-Stass A., O’Donovan L., Lehto T., Garcia-Guerra A., Nordin J. (2015). Self-Assembly into Nanoparticles Is Essential for Receptor Mediated Uptake of Therapeutic Antisense Oligonucleotides. Nano Lett..

[94] Boutorin A.S., Gus’kova L.V., Ivanova E.M., Kobetz N.D., Zarytova V.F., Ryte A.S., Yurchenko L.V., Vlassov V.V. (1989). Synthesis of alkylating oligonucleotide derivatives containing cholesterol or phenazinium residues at their 3′-terminus and their interaction with DNA within mammalian cells. FEBS Lett..

[95] Li X., Chen Y., Wang M., Ma Y., Xia W., Gu H. (2013). A mesoporous silica nanoparticle—EI—Fusogenic peptide system for siRNA delivery in cancer therapy. Biomaterials.

[96] Rupaimoole R., Slack F.J. (2017). MicroRNA therapeutics: Towards a new era for the management of cancer and other diseases. Nat. Rev. Drug Discov..

[97] Ferguson C.M., Echeverria D., Hassler M., Ly S., Khvorova A. (2020). Cell Type Impacts Accessibility of mRNA to Silencing by RNA Interference. Mol. Ther. Nucleic.

[98] de Fougerolles A., Vornlocher H.-P., Maraganore J., Lieberman J. (2007). Interfering with disease: A progress report on siRNA-based therapeutics. Nat. Rev. Drug Discov..

[99] Watts J.K., Corey D.R. (2012). Silencing disease genes in the laboratory and the clinic. J. Pathol..

[100] Zhu S., Zhang T., Zheng L., Liu H., Song W., Liu D., Li Z., Pan C.-x. (2021). Combination strategies to maximize the benefits of cancer immunotherapy. J. Hematol. Oncol..

[101] Mokhtari R.B., Homayouni T.S., Baluch N., Morgatskaya E., Kumar S., Das B., Yeger H. (2017). Combination therapy in combating cancer. Oncotarget.

[102] Kutova O.M., Guryev E.L., Sokolova E.A., Alzeibak R., Balalaeva I.V. (2019). Targeted Delivery to Tumors: Multidirectional Strategies to Improve Treatment Efficiency. Cancers.

[103] Shin M.D., Shukla S., Chung Y.H., Beiss V., Chan S.K., Ortega-Rivera O.A., Wirth D.M., Chen A., Sack M., Pokorski J.K. (2020). COVID-19 vaccine development and a potential nanomaterial path forward. Nat. Nanotechnol..

[104] Ojasalo S., Piskunen P., Shen B., Kostiainen M.A., Linko V. (2021). Hybrid Nanoassemblies from Viruses and DNA Nanostructures. Nanomaterials.

[105] Tian T., Li Y., Lin Y. (2022). Prospects and challenges of dynamic DNA nanostructures in biomedical applications. Bone Res..

[106] Zimmerman M.I., Porter J.R., Ward M.D., Singh S., Vithani N., Meller A., Mallimadugula U.L., Kuhn C.E., Borowsky J.H., Wiewiora R.P. (2021). SARS-CoV-2 simulations go exascale to predict dramatic spike opening and cryptic pockets across the proteome. Nat. Chem..

[107] Guevara M.L., Persano F., Persano S. (2020). Advances in Lipid Nanoparticles for mRNA-Based Cancer Immunotherapy. Front. Chem..

[108] Hou X., Zaks T., Langer R., Dong Y. (2021). Lipid nanoparticles for mRNA delivery. Nat. Rev. Mater..

[109] Tenchov R., Bird R., Curtze A.E., Zhou Q. (2021). Lipid Nanoparticles─From Liposomes to mRNA Vaccine Delivery, a Landscape of Research Diversity and Advancement. ACS Nano.

[110] Pardi N., Hogan M.J., Porter F.W., Weissman D. (2018). mRNA vaccines—A new era in vaccinology. Nat. Rev. Drug Discov..

